# Supplementary material for: HIVprotI: an integrated web based platform for prediction and design of HIV proteins inhibitors
Source: J Cheminform. 2018 Mar 9;10:12. doi: 10.1186/s13321-018-0266-y (PMC5845081; doi:10.1186/s13321-018-0266-y)
Supplement: Supplementary file 2 — Additional file 2. Source code of HIVProtI web server. [file 13321_2018_266_MOESM2_ESM.zip › HIVprotI_Source-code/hivprotI/weka-3-6-6/doc/weka/core/Utils.html]

Utils


---


|  |  |  |
| --- | --- | --- |
| **Overview** \| **Package** \| **Class** \| **Tree** \| **Deprecated** \| **Index** \| **Help** \| **Weka's home** | |  |
| **PREV CLASS**   **NEXT CLASS** | **FRAMES**    **NO FRAMES**     **All Classes** |  |
| SUMMARY: NESTED \| FIELD \| CONSTR \| METHOD | DETAIL: FIELD \| CONSTR \| METHOD |  |


---


## weka.core Class Utils

```
java.lang.Object
  weka.core.Utils
```

**All Implemented Interfaces:**: RevisionHandler

---

``` public final class Utils extends java.lang.Object implements RevisionHandler ```

Class implementing some simple utility methods.

**Version:**
:   $Revision: 7553 $

**Author:**
:   Eibe Frank, Yong Wang, Len Trigg, Julien Prados

---

| **Field Summary** | |
| --- | --- |
| `static double` | `log2`             The natural logarithm of 2. |
| `static double` | `SMALL`             The small deviation allowed in double comparisons. |


**Constructor Summary**

`Utils()`


| **Method Summary** | |
| --- | --- |
| `static java.lang.String` | `arrayToString(java.lang.Object array)`             Returns the given Array in a string representation. |
| `static java.lang.String` | `backQuoteChars(java.lang.String string)`             Converts carriage returns and new lines in a string into \r and \n. |
| `static void` | `checkForRemainingOptions(java.lang.String[] options)`             Checks if the given array contains any non-empty options. |
| `static java.lang.String` | `convertNewLines(java.lang.String string)`             Converts carriage returns and new lines in a string into \r and \n. |
| `static java.io.File` | `convertToRelativePath(java.io.File absolute)`             Converts a File's absolute path to a path relative to the user (ie start) directory. |
| `static double` | `correlation(double[] y1, double[] y2, int n)`             Returns the correlation coefficient of two double vectors. |
| `static java.lang.String` | `doubleToString(double value, int afterDecimalPoint)`             Rounds a double and converts it into String. |
| `static java.lang.String` | `doubleToString(double value, int width, int afterDecimalPoint)`             Rounds a double and converts it into a formatted decimal-justified String. |
| `static boolean` | `eq(double a, double b)`             Tests if a is equal to b. |
| `static java.lang.Object` | `forName(java.lang.Class classType, java.lang.String className, java.lang.String[] options)`             Creates a new instance of an object given it's class name and (optional) arguments to pass to it's setOptions method. |
| `static java.lang.Class` | `getArrayClass(java.lang.Class c)`             Returns the basic class of an array class (handles multi-dimensional arrays). |
| `static int` | `getArrayDimensions(java.lang.Class array)`             Returns the dimensions of the given array. |
| `static int` | `getArrayDimensions(java.lang.Object array)`             Returns the dimensions of the given array. |
| `static boolean` | `getFlag(char flag, java.lang.String[] options)`             Checks if the given array contains the flag "-Char". |
| `static boolean` | `getFlag(java.lang.String flag, java.lang.String[] options)`             Checks if the given array contains the flag "-String". |
| `static java.lang.String` | `getOption(char flag, java.lang.String[] options)`             Gets an option indicated by a flag "-Char" from the given array of strings. |
| `static java.lang.String` | `getOption(java.lang.String flag, java.lang.String[] options)`             Gets an option indicated by a flag "-String" from the given array of strings. |
| `static int` | `getOptionPos(char flag, java.lang.String[] options)`             Gets the index of an option or flag indicated by a flag "-Char" from the given array of strings. |
| `static int` | `getOptionPos(java.lang.String flag, java.lang.String[] options)`             Gets the index of an option or flag indicated by a flag "-String" from the given array of strings. |
| `java.lang.String` | `getRevision()`             Returns the revision string. |
| `static boolean` | `gr(double a, double b)`             Tests if a is greater than b. |
| `static boolean` | `grOrEq(double a, double b)`             Tests if a is greater or equal to b. |
| `static double` | `info(int[] counts)`             Computes entropy for an array of integers. |
| `static java.lang.String` | `joinOptions(java.lang.String[] optionArray)`             Joins all the options in an option array into a single string, as might be used on the command line. |
| `static double` | `kthSmallestValue(double[] array, int k)`             Returns the kth-smallest value in the array |
| `static double` | `kthSmallestValue(int[] array, int k)`             Returns the kth-smallest value in the array. |
| `static double` | `log2(double a)`             Returns the logarithm of a for base 2. |
| `static double[]` | `logs2probs(double[] a)`             Converts an array containing the natural logarithms of probabilities stored in a vector back into probabilities. |
| `static void` | `main(java.lang.String[] ops)`             Main method for testing this class. |
| `static int` | `maxIndex(double[] doubles)`             Returns index of maximum element in a given array of doubles. |
| `static int` | `maxIndex(int[] ints)`             Returns index of maximum element in a given array of integers. |
| `static double` | `mean(double[] vector)`             Computes the mean for an array of doubles. |
| `static int` | `minIndex(double[] doubles)`             Returns index of minimum element in a given array of doubles. |
| `static int` | `minIndex(int[] ints)`             Returns index of minimum element in a given array of integers. |
| `static void` | `normalize(double[] doubles)`             Normalizes the doubles in the array by their sum. |
| `static void` | `normalize(double[] doubles, double sum)`             Normalizes the doubles in the array using the given value. |
| `static java.lang.String` | `padLeft(java.lang.String inString, int length)`             Pads a string to a specified length, inserting spaces on the left as required. |
| `static java.lang.String` | `padRight(java.lang.String inString, int length)`             Pads a string to a specified length, inserting spaces on the right as required. |
| `static java.lang.String[]` | `partitionOptions(java.lang.String[] options)`             Returns the secondary set of options (if any) contained in the supplied options array. |
| `static int` | `probRound(double value, java.util.Random rand)`             Rounds a double to the next nearest integer value in a probabilistic fashion (e.g. |
| `static double` | `probToLogOdds(double prob)`             Returns the log-odds for a given probabilitiy. |
| `static java.lang.String` | `quote(java.lang.String string)`             Quotes a string if it contains special characters. |
| `static java.util.Properties` | `readProperties(java.lang.String resourceName)`             Reads properties that inherit from three locations. |
| `static java.lang.String` | `removeSubstring(java.lang.String inString, java.lang.String substring)`             Removes all occurrences of a string from another string. |
| `static java.lang.String` | `replaceSubstring(java.lang.String inString, java.lang.String subString, java.lang.String replaceString)`             Replaces with a new string, all occurrences of a string from another string. |
| `static java.lang.String` | `revertNewLines(java.lang.String string)`             Reverts \r and \n in a string into carriage returns and new lines. |
| `static int` | `round(double value)`             Rounds a double to the next nearest integer value. |
| `static double` | `roundDouble(double value, int afterDecimalPoint)`             Rounds a double to the given number of decimal places. |
| `static boolean` | `sm(double a, double b)`             Tests if a is smaller than b. |
| `static boolean` | `smOrEq(double a, double b)`             Tests if a is smaller or equal to b. |
| `static int[]` | `sort(double[] array)`             Sorts a given array of doubles in ascending order and returns an array of integers with the positions of the elements of the original array in the sorted array. |
| `static int[]` | `sort(int[] array)`             Sorts a given array of integers in ascending order and returns an array of integers with the positions of the elements of the original array in the sorted array. |
| `static java.lang.String[]` | `splitOptions(java.lang.String quotedOptionString)`             Split up a string containing options into an array of strings, one for each option. |
| `static int[]` | `stableSort(double[] array)`             Sorts a given array of doubles in ascending order and returns an array of integers with the positions of the elements of the original array in the sorted array. |
| `static double` | `sum(double[] doubles)`             Computes the sum of the elements of an array of doubles. |
| `static int` | `sum(int[] ints)`             Computes the sum of the elements of an array of integers. |
| `static java.lang.String` | `unbackQuoteChars(java.lang.String string)`             The inverse operation of backQuoteChars(). |
| `static java.lang.String` | `unquote(java.lang.String string)`             unquotes are previously quoted string (but only if necessary), i.e., it removes the single quotes around it. |
| `static double` | `variance(double[] vector)`             Computes the variance for an array of doubles. |
| `static double` | `xlogx(int c)`             Returns c\*log2(c) for a given integer value c. |

**Methods inherited from class java.lang.Object**

`equals, getClass, hashCode, notify, notifyAll, toString, wait, wait, wait`

**Field Detail**

### log2

```
public static double log2
```

:   The natural logarithm of 2.

---


### SMALL

```
public static double SMALL
```

:   The small deviation allowed in double comparisons.


**Constructor Detail**

### Utils

```
public Utils()
```


**Method Detail**

### readProperties

```
public static java.util.Properties readProperties(java.lang.String resourceName)
                                           throws java.lang.Exception
```

:   Reads properties that inherit from three locations. Properties
    are first defined in the system resource location (i.e. in the
    CLASSPATH). These default properties must exist. Properties
    defined in the users home directory (optional) override default
    settings. Properties defined in the current directory (optional)
    override all these settings.

    :   **Parameters:**: `resourceName` - the location of the resource that should be loaded. e.g.: "weka/core/Utils.props". (The use of hardcoded forward slashes here is OK - see jdk1.1/docs/guide/misc/resources.html) This routine will also look for the file (in this case) "Utils.props" in the users home directory and the current directory. **Returns:**: the Properties **Throws:**: `java.lang.Exception` - if no default properties are defined, or if an error occurs reading the properties files.

---


### correlation

```
public static final double correlation(double[] y1,
                                       double[] y2,
                                       int n)
```

:   Returns the correlation coefficient of two double vectors.

    :   **Parameters:**: `y1` - double vector 1: `y2` - double vector 2: `n` - the length of two double vectors **Returns:**: the correlation coefficient

---


### removeSubstring

```
public static java.lang.String removeSubstring(java.lang.String inString,
                                               java.lang.String substring)
```

:   Removes all occurrences of a string from another string.

    :   **Parameters:**: `inString` - the string to remove substrings from.: `substring` - the substring to remove. **Returns:**: the input string with occurrences of substring removed.

---


### replaceSubstring

```
public static java.lang.String replaceSubstring(java.lang.String inString,
                                                java.lang.String subString,
                                                java.lang.String replaceString)
```

:   Replaces with a new string, all occurrences of a string from
    another string.

    :   **Parameters:**: `inString` - the string to replace substrings in.: `subString` - the substring to replace.: `replaceString` - the replacement substring **Returns:**: the input string with occurrences of substring replaced.

---


### padLeft

```
public static java.lang.String padLeft(java.lang.String inString,
                                       int length)
```

:   Pads a string to a specified length, inserting spaces on the left
    as required. If the string is too long, characters are removed (from
    the right).

    :   **Parameters:**: `inString` - the input string: `length` - the desired length of the output string **Returns:**: the output string

---


### padRight

```
public static java.lang.String padRight(java.lang.String inString,
                                        int length)
```

:   Pads a string to a specified length, inserting spaces on the right
    as required. If the string is too long, characters are removed (from
    the right).

    :   **Parameters:**: `inString` - the input string: `length` - the desired length of the output string **Returns:**: the output string

---


### doubleToString

```
public static java.lang.String doubleToString(double value,
                                              int afterDecimalPoint)
```

:   Rounds a double and converts it into String.

    :   **Parameters:**: `value` - the double value: `afterDecimalPoint` - the (maximum) number of digits permitted after the decimal point **Returns:**: the double as a formatted string

---


### doubleToString

```
public static java.lang.String doubleToString(double value,
                                              int width,
                                              int afterDecimalPoint)
```

:   Rounds a double and converts it into a formatted decimal-justified String.
    Trailing 0's are replaced with spaces.

    :   **Parameters:**: `value` - the double value: `width` - the width of the string: `afterDecimalPoint` - the number of digits after the decimal point **Returns:**: the double as a formatted string

---


### getArrayClass

```
public static java.lang.Class getArrayClass(java.lang.Class c)
```

:   Returns the basic class of an array class (handles multi-dimensional
    arrays).

    :   **Parameters:**: `c` - the array to inspect **Returns:**: the class of the innermost elements

---


### getArrayDimensions

```
public static int getArrayDimensions(java.lang.Class array)
```

:   Returns the dimensions of the given array. Even though the
    parameter is of type "Object" one can hand over primitve arrays, e.g.
    int[3] or double[2][4].

    :   **Parameters:**: `array` - the array to determine the dimensions for **Returns:**: the dimensions of the array

---


### getArrayDimensions

```
public static int getArrayDimensions(java.lang.Object array)
```

:   Returns the dimensions of the given array. Even though the
    parameter is of type "Object" one can hand over primitve arrays, e.g.
    int[3] or double[2][4].

    :   **Parameters:**: `array` - the array to determine the dimensions for **Returns:**: the dimensions of the array

---


### arrayToString

```
public static java.lang.String arrayToString(java.lang.Object array)
```

:   Returns the given Array in a string representation. Even though the
    parameter is of type "Object" one can hand over primitve arrays, e.g.
    int[3] or double[2][4].

    :   **Parameters:**: `array` - the array to return in a string representation **Returns:**: the array as string

---


### eq

```
public static boolean eq(double a,
                         double b)
```

:   Tests if a is equal to b.

    :   **Parameters:**: `a` - a double: `b` - a double

---


### checkForRemainingOptions

```
public static void checkForRemainingOptions(java.lang.String[] options)
                                     throws java.lang.Exception
```

:   Checks if the given array contains any non-empty options.

    :   **Parameters:**: `options` - an array of strings **Throws:**: `java.lang.Exception` - if there are any non-empty options

---


### getFlag

```
public static boolean getFlag(char flag,
                              java.lang.String[] options)
                       throws java.lang.Exception
```

:   Checks if the given array contains the flag "-Char". Stops
    searching at the first marker "--". If the flag is found,
    it is replaced with the empty string.

    :   **Parameters:**: `flag` - the character indicating the flag.: `options` - the array of strings containing all the options. **Returns:**: true if the flag was found **Throws:**: `java.lang.Exception` - if an illegal option was found

---


### getFlag

```
public static boolean getFlag(java.lang.String flag,
                              java.lang.String[] options)
                       throws java.lang.Exception
```

:   Checks if the given array contains the flag "-String". Stops
    searching at the first marker "--". If the flag is found,
    it is replaced with the empty string.

    :   **Parameters:**: `flag` - the String indicating the flag.: `options` - the array of strings containing all the options. **Returns:**: true if the flag was found **Throws:**: `java.lang.Exception` - if an illegal option was found

---


### getOption

```
public static java.lang.String getOption(char flag,
                                         java.lang.String[] options)
                                  throws java.lang.Exception
```

:   Gets an option indicated by a flag "-Char" from the given array
    of strings. Stops searching at the first marker "--". Replaces
    flag and option with empty strings.

    :   **Parameters:**: `flag` - the character indicating the option.: `options` - the array of strings containing all the options. **Returns:**: the indicated option or an empty string **Throws:**: `java.lang.Exception` - if the option indicated by the flag can't be found

---


### getOption

```
public static java.lang.String getOption(java.lang.String flag,
                                         java.lang.String[] options)
                                  throws java.lang.Exception
```

:   Gets an option indicated by a flag "-String" from the given array
    of strings. Stops searching at the first marker "--". Replaces
    flag and option with empty strings.

    :   **Parameters:**: `flag` - the String indicating the option.: `options` - the array of strings containing all the options. **Returns:**: the indicated option or an empty string **Throws:**: `java.lang.Exception` - if the option indicated by the flag can't be found

---


### getOptionPos

```
public static int getOptionPos(char flag,
                               java.lang.String[] options)
```

:   Gets the index of an option or flag indicated by a flag "-Char" from
    the given array of strings. Stops searching at the first marker "--".

    :   **Parameters:**: `flag` - the character indicating the option.: `options` - the array of strings containing all the options. **Returns:**: the position if found, or -1 otherwise

---


### getOptionPos

```
public static int getOptionPos(java.lang.String flag,
                               java.lang.String[] options)
```

:   Gets the index of an option or flag indicated by a flag "-String" from
    the given array of strings. Stops searching at the first marker "--".

    :   **Parameters:**: `flag` - the String indicating the option.: `options` - the array of strings containing all the options. **Returns:**: the position if found, or -1 otherwise

---


### quote

```
public static java.lang.String quote(java.lang.String string)
```

:   Quotes a string if it contains special characters.
    The following rules are applied:
    A character is backquoted version of it is one
    of " ' % \ \n \r \t.
    A string is enclosed within single quotes if a character has been
    backquoted using the previous rule above or contains
    { } or is exactly equal to the strings
    , ? space or "" (empty string).
    A quoted question mark distinguishes it from the missing value which
    is represented as an unquoted question mark in arff files.

    :   **Parameters:**: `string` - the string to be quoted **Returns:**: the string (possibly quoted) **See Also:**: `unquote(String)`

---


### unquote

```
public static java.lang.String unquote(java.lang.String string)
```

:   unquotes are previously quoted string (but only if necessary), i.e., it
    removes the single quotes around it. Inverse to quote(String).

    :   **Parameters:**: `string` - the string to process **Returns:**: the unquoted string **See Also:**: `quote(String)`

---


### backQuoteChars

```
public static java.lang.String backQuoteChars(java.lang.String string)
```

:   Converts carriage returns and new lines in a string into \r and \n.
    Backquotes the following characters: ` " \ \t and %

    :   **Parameters:**: `string` - the string **Returns:**: the converted string **See Also:**: `unbackQuoteChars(String)`

---


### convertNewLines

```
public static java.lang.String convertNewLines(java.lang.String string)
```

:   Converts carriage returns and new lines in a string into \r and \n.

    :   **Parameters:**: `string` - the string **Returns:**: the converted string

---


### revertNewLines

```
public static java.lang.String revertNewLines(java.lang.String string)
```

:   Reverts \r and \n in a string into carriage returns and new lines.

    :   **Parameters:**: `string` - the string **Returns:**: the converted string

---


### partitionOptions

```
public static java.lang.String[] partitionOptions(java.lang.String[] options)
```

:   Returns the secondary set of options (if any) contained in
    the supplied options array. The secondary set is defined to
    be any options after the first "--". These options are removed from
    the original options array.

    :   **Parameters:**: `options` - the input array of options **Returns:**: the array of secondary options

---


### unbackQuoteChars

```
public static java.lang.String unbackQuoteChars(java.lang.String string)
```

:   The inverse operation of backQuoteChars().
    Converts back-quoted carriage returns and new lines in a string
    to the corresponding character ('\r' and '\n').
    Also "un"-back-quotes the following characters: ` " \ \t and %

    :   **Parameters:**: `string` - the string **Returns:**: the converted string **See Also:**: `backQuoteChars(String)`

---


### splitOptions

```
public static java.lang.String[] splitOptions(java.lang.String quotedOptionString)
                                       throws java.lang.Exception
```

:   Split up a string containing options into an array of strings,
    one for each option.

    :   **Parameters:**: `quotedOptionString` - the string containing the options **Returns:**: the array of options **Throws:**: `java.lang.Exception` - in case of an unterminated string, unknown character or a parse error

---


### joinOptions

```
public static java.lang.String joinOptions(java.lang.String[] optionArray)
```

:   Joins all the options in an option array into a single string,
    as might be used on the command line.

    :   **Parameters:**: `optionArray` - the array of options **Returns:**: the string containing all options.

---


### forName

```
public static java.lang.Object forName(java.lang.Class classType,
                                       java.lang.String className,
                                       java.lang.String[] options)
                                throws java.lang.Exception
```

:   Creates a new instance of an object given it's class name and
    (optional) arguments to pass to it's setOptions method. If the
    object implements OptionHandler and the options parameter is
    non-null, the object will have it's options set. Example use:

    ```` ```
     String classifierName = Utils.getOption('W', options);
     Classifier c = (Classifier)Utils.forName(Classifier.class,
                                              classifierName,
                                              options);
     setClassifier(c);
    ``` ```` 

    :   **Parameters:**: `classType` - the class that the instantiated object should be assignable to -- an exception is thrown if this is not the case: `className` - the fully qualified class name of the object: `options` - an array of options suitable for passing to setOptions. May be null. Any options accepted by the object will be removed from the array. **Returns:**: the newly created object, ready for use. **Throws:**: `java.lang.Exception` - if the class name is invalid, or if the class is not assignable to the desired class type, or the options supplied are not acceptable to the object

---


### info

```
public static double info(int[] counts)
```

:   Computes entropy for an array of integers.

    :   **Parameters:**: `counts` - array of counts **Returns:**: - a log2 a - b log2 b - c log2 c + (a+b+c) log2 (a+b+c) when given array [a b c]

---


### smOrEq

```
public static boolean smOrEq(double a,
                             double b)
```

:   Tests if a is smaller or equal to b.

    :   **Parameters:**: `a` - a double: `b` - a double

---


### grOrEq

```
public static boolean grOrEq(double a,
                             double b)
```

:   Tests if a is greater or equal to b.

    :   **Parameters:**: `a` - a double: `b` - a double

---


### sm

```
public static boolean sm(double a,
                         double b)
```

:   Tests if a is smaller than b.

    :   **Parameters:**: `a` - a double: `b` - a double

---


### gr

```
public static boolean gr(double a,
                         double b)
```

:   Tests if a is greater than b.

    :   **Parameters:**: `a` - a double: `b` - a double

---


### kthSmallestValue

```
public static double kthSmallestValue(int[] array,
                                      int k)
```

:   Returns the kth-smallest value in the array.

    :   **Parameters:**: `array` - the array of integers: `k` - the value of k **Returns:**: the kth-smallest value

---


### kthSmallestValue

```
public static double kthSmallestValue(double[] array,
                                      int k)
```

:   Returns the kth-smallest value in the array

    :   **Parameters:**: `array` - the array of double: `k` - the value of k **Returns:**: the kth-smallest value

---


### log2

```
public static double log2(double a)
```

:   Returns the logarithm of a for base 2.

    :   **Parameters:**: `a` - a double **Returns:**: the logarithm for base 2

---


### maxIndex

```
public static int maxIndex(double[] doubles)
```

:   Returns index of maximum element in a given
    array of doubles. First maximum is returned.

    :   **Parameters:**: `doubles` - the array of doubles **Returns:**: the index of the maximum element

---


### maxIndex

```
public static int maxIndex(int[] ints)
```

:   Returns index of maximum element in a given
    array of integers. First maximum is returned.

    :   **Parameters:**: `ints` - the array of integers **Returns:**: the index of the maximum element

---


### mean

```
public static double mean(double[] vector)
```

:   Computes the mean for an array of doubles.

    :   **Parameters:**: `vector` - the array **Returns:**: the mean

---


### minIndex

```
public static int minIndex(int[] ints)
```

:   Returns index of minimum element in a given
    array of integers. First minimum is returned.

    :   **Parameters:**: `ints` - the array of integers **Returns:**: the index of the minimum element

---


### minIndex

```
public static int minIndex(double[] doubles)
```

:   Returns index of minimum element in a given
    array of doubles. First minimum is returned.

    :   **Parameters:**: `doubles` - the array of doubles **Returns:**: the index of the minimum element

---


### normalize

```
public static void normalize(double[] doubles)
```

:   Normalizes the doubles in the array by their sum.

    :   **Parameters:**: `doubles` - the array of double **Throws:**: `java.lang.IllegalArgumentException` - if sum is Zero or NaN

---


### normalize

```
public static void normalize(double[] doubles,
                             double sum)
```

:   Normalizes the doubles in the array using the given value.

    :   **Parameters:**: `doubles` - the array of double: `sum` - the value by which the doubles are to be normalized **Throws:**: `java.lang.IllegalArgumentException` - if sum is zero or NaN

---


### logs2probs

```
public static double[] logs2probs(double[] a)
```

:   Converts an array containing the natural logarithms of
    probabilities stored in a vector back into probabilities.
    The probabilities are assumed to sum to one.

    :   **Parameters:**: `a` - an array holding the natural logarithms of the probabilities **Returns:**: the converted array

---


### probToLogOdds

```
public static double probToLogOdds(double prob)
```

:   Returns the log-odds for a given probabilitiy.

    :   **Parameters:**: `prob` - the probabilitiy **Returns:**: the log-odds after the probability has been mapped to [Utils.SMALL, 1-Utils.SMALL]

---


### round

```
public static int round(double value)
```

:   Rounds a double to the next nearest integer value. The JDK version
    of it doesn't work properly.

    :   **Parameters:**: `value` - the double value **Returns:**: the resulting integer value

---


### probRound

```
public static int probRound(double value,
                            java.util.Random rand)
```

:   Rounds a double to the next nearest integer value in a probabilistic
    fashion (e.g. 0.8 has a 20% chance of being rounded down to 0 and a
    80% chance of being rounded up to 1). In the limit, the average of
    the rounded numbers generated by this procedure should converge to
    the original double.

    :   **Parameters:**: `value` - the double value: `rand` - the random number generator **Returns:**: the resulting integer value

---


### roundDouble

```
public static double roundDouble(double value,
                                 int afterDecimalPoint)
```

:   Rounds a double to the given number of decimal places.

    :   **Parameters:**: `value` - the double value: `afterDecimalPoint` - the number of digits after the decimal point **Returns:**: the double rounded to the given precision

---


### sort

```
public static int[] sort(int[] array)
```

:   Sorts a given array of integers in ascending order and returns an
    array of integers with the positions of the elements of the original
    array in the sorted array. The sort is stable. (Equal elements remain
    in their original order.)

    :   **Parameters:**: `array` - this array is not changed by the method! **Returns:**: an array of integers with the positions in the sorted array.

---


### sort

```
public static int[] sort(double[] array)
```

:   Sorts a given array of doubles in ascending order and returns an
    array of integers with the positions of the elements of the
    original array in the sorted array. NOTE THESE CHANGES: the sort
    is no longer stable and it doesn't use safe floating-point
    comparisons anymore. Occurrences of Double.NaN are treated as
    Double.MAX\_VALUE

    :   **Parameters:**: `array` - this array is not changed by the method! **Returns:**: an array of integers with the positions in the sorted array.

---


### stableSort

```
public static int[] stableSort(double[] array)
```

:   Sorts a given array of doubles in ascending order and returns an
    array of integers with the positions of the elements of the original
    array in the sorted array. The sort is stable (Equal elements remain
    in their original order.) Occurrences of Double.NaN are treated as
    Double.MAX\_VALUE

    :   **Parameters:**: `array` - this array is not changed by the method! **Returns:**: an array of integers with the positions in the sorted array.

---


### variance

```
public static double variance(double[] vector)
```

:   Computes the variance for an array of doubles.

    :   **Parameters:**: `vector` - the array **Returns:**: the variance

---


### sum

```
public static double sum(double[] doubles)
```

:   Computes the sum of the elements of an array of doubles.

    :   **Parameters:**: `doubles` - the array of double **Returns:**: the sum of the elements

---


### sum

```
public static int sum(int[] ints)
```

:   Computes the sum of the elements of an array of integers.

    :   **Parameters:**: `ints` - the array of integers **Returns:**: the sum of the elements

---


### xlogx

```
public static double xlogx(int c)
```

:   Returns c\*log2(c) for a given integer value c.

    :   **Parameters:**: `c` - an integer value **Returns:**: c\*log2(c) (but is careful to return 0 if c is 0)

---


### convertToRelativePath

```
public static java.io.File convertToRelativePath(java.io.File absolute)
                                          throws java.lang.Exception
```

:   Converts a File's absolute path to a path relative to the user
    (ie start) directory. Includes an additional workaround for Cygwin, which
    doesn't like upper case drive letters.

    :   **Parameters:**: `absolute` - the File to convert to relative path **Returns:**: a File with a path that is relative to the user's directory **Throws:**: `java.lang.Exception` - if the path cannot be constructed

---


### getRevision

```
public java.lang.String getRevision()
```

:   Returns the revision string.

    :   **Specified by:**: `getRevision` in interface `RevisionHandler`
    :   **Returns:**: the revision

---


### main

```
public static void main(java.lang.String[] ops)
```

:   Main method for testing this class.

    :   **Parameters:**: `ops` - some dummy options


---


|  |  |  |
| --- | --- | --- |
| **Overview** \| **Package** \| **Class** \| **Tree** \| **Deprecated** \| **Index** \| **Help** \| **Weka's home** | |  |
| **PREV CLASS**   **NEXT CLASS** | **FRAMES**    **NO FRAMES**     **All Classes** |  |
| SUMMARY: NESTED \| FIELD \| CONSTR \| METHOD | DETAIL: FIELD \| CONSTR \| METHOD |  |


---
